# Cognitive control in music: adaptive strategies for relative pitch across the absolute-pitch proficiency continuum

**DOI:** 10.3389/fpsyg.2025.1723224

**Published:** 2026-01-09

**Authors:** Karen Shibayama, Hitoshi Shimada, Kosuke Itoh

**Affiliations:** Center for Integrated Human Brain Science, Brain Research Institute, Niigata University, Niigata, Japan

**Keywords:** absolute pitch, cognitive strategy, music cognition, relative pitch, solfege

## Abstract

Absolute pitch (AP) is often regarded as a rare gift, yet Western tonal music relies more on relative pitch (RP), which encodes meaning through intervals to the keynote (tonic). This contrast offers a natural test bed for cognitive control: AP functions as an automatic, stimulus-bound code, whereas RP demands context-dependent computation. We tested 50 non–music-major students spanning the AP continuum on a movable-Do solfa-naming task under three tonal contexts of increasing difficulty (C major, B major, randomly shifting keys). AP conferred overall advantage, but error patterns revealed adaptive strategy shifts: (i) direct AP use in C major, (ii) transposition of pitch names in B major, and (iii) chord-component listening in random keys, with individual variability. Non-AP participants relied consistently on RP. Thus, scale-note identification involves flexible strategy selection shaped by AP proficiency and tonal context, demonstrating cognitive control in a naturalistic musical setting and motivating tailored ear-training in education.

## Introduction

Cognitive control theories propose that the brain flexibly selects among competing representations and strategies to optimize performance as task demands change (Botvinick et al., 2001; Shenhav et al., 2013; Millera and Cohen, 2001). Musical pitch processing offers a naturalistic window into this meta-decision process, as different listeners rely to different degrees on two qualitatively distinct coding systems: absolute pitch (AP) and relative pitch (RP).

AP is the ability to name or reproduce a pitch in isolation without an external reference, supporting rapid and automatic stimulus-driven label retrieval (e.g., “440 Hz → A”) (Schulze et al., 2013; Levitin and Rogers, 2005). Extensive research has established AP as a form of auditory expertise: pitch labeling with AP constitutes an absolute sensory judgment that is fast, automatic, and seemingly effortless (Takeuchi and Hulse, 1993; Di Stefano and Spence, 2024). Neurobiologically, this ability is associated with specialized circuitry in the auditory association cortex and dorsolateral prefrontal cortex (e.g., Elmer et al., 2015; Itoh et al., 2005; Keenan et al., 2001; Matsuda et al., 2019; Oechslin et al., 2010; Schlaug et al., 1995; Wilson et al., 2009; Wengenroth et al., 2014; Zatorre et al., 1998), although the precise mechanisms remain to be fully delineated (Kim and Knösche, 2017; Zatorre, 2003).

In contrast, RP requires context-dependent computations over intervallic structure to determine how far apart two notes are or which scale degree (do, re, mi, etc.) a tone represents (Deutsch, 2013; Itoh et al., 2005; Takeuchi and Hulse, 1993; Zatorre, 2003). Pitch naming via RP is slower and less automatic because it necessitates comparing the heard pitch against a reference maintained in short-term auditory memory. This distinction was demonstrated in Itoh et al. (2005), where RP listeners showed slower pitch-naming responses than AP listeners, and in Miyazaki’s (1995) finding that AP possessors exhibit longer reaction times when naming out-of-tune tones, which cannot be labeled using AP and instead require RP computations. It is important to differentiate this form of RP-based pitch naming from the automatic detection of out-of-key notes within a tonal sequence, as examined by Brattico et al. (2006). Even non-AP listeners can preattentively detect tonal violations, but identifying the specific pitch name demands controlled processing supported by working memory. Although relatively few studies have probed the neural mechanisms underlying RP-based pitch naming, Itoh et al. (2005) showed that it recruits more spatially distributed and temporally extended neural activity than the rapid, automatic responses associated with AP. These findings support the view that RP-based pitch naming depends on controlled processes governed by frontal-lobe functions.

Accordingly, AP and RP instantiate two competing codes, one fast and stimulus-bound “fixed-Do” labeling and the other context-dependent “movable-Do” labeling, which mirror the automatic vs. controlled representations that cognitive-control theories posit across domains. In Western tonal music, the core of musical meaning and structural understanding lies not in isolated notes, but in the relative pitch relationships that form melodies and harmonies and in their functional roles within scales and keys. This is because individual notes carry little inherent musical information on their own; their significance emerges from how they relate to surrounding tones, such as forming intervals, outlining chords, or establishing expectations within a tonal hierarchy (Krumhansl, 1990; Piston and DeVoto, 1987). These relational patterns allow listeners to perceive key, recognize harmonic progressions, anticipate resolutions, and experience tension and release, all of which make RP foundational to musical comprehension in the Western tonal system. Nevertheless, a substantial minority of musicians possess AP, creating an intrinsic representational conflict that demands strategic control where RP is important.

Behavioral evidence suggests that this conflict is not always resolved efficiently: AP musicians often slow down or make errors when melodies are transposed, sight-reading involves key changes, or scale degrees must be identified using moveable-Do solmization (Miyazaki and Rakowski, 2002; Wilson et al., 2009; Mito, 2003). These difficulties resemble classic Stroop or flanker interference effects (Stroop, 1935; Schulze et al., 2013; Eriksen and Eriksen, 1974; Sharma et al., 2021), in which dominant responses must be inhibited or transformed. Indeed, listeners with AP show Stroop-like interference when a pitch is sung with an incongruent pitch name, when, for example, the pitch of D is sung with the syllable “Do”, which is consistent with the key of D in the movable-Do system (Itoh et al., 2005). On the other hand, other studies report no such disadvantage (Temperley and Marvin, 2008; Greber and Jäncke, 2020), suggesting that some AP musicians may exhibit latent flexibility, such as mental transposition or selective attention to interval cues. A key limitation of prior research is its reliance on group averages, which obscure the diversity of strategies within the AP population. Another limitation concerned the difficulty in depicting the dynamic process of how cognitive control selects the appropriate strategy among alternatives.

Accordingly, the present study used error-pattern and cluster analyses to reveal how cognitive control operates across the AP continuum during an RP task of varying difficulty. Fifty non-music-major undergraduates with a wide range of AP proficiency used the movable-Do system to name a tone that followed authentic cadences under three progressively demanding tonal contexts: (i) fixed C major (minimal conflict), (ii) fixed B major (systematic and predictable conflict), and (iii) randomly shifting major keys (maximal conflict and uncertainty). Like the Stroop, Simon, or flanker tasks, a key change created a representational conflict that required suppression or transformation of the automatic and dominant fixed-Do labels. Randomization of key further increased the cognitive demand by necessitating trial-by-trial reframing of pitch-name mapping from fixed-Do to movable-Do. By integrating accuracy, reaction time, and confusion-matrix structure, we traced when listeners relied on fixed AP strategies, performed on-the-fly pitch-name transpositions, or shifted to interval-based or alternative strategies.

Our central question is thus not whether AP helps or hinders RP, as previous music cognition studies have framed it, but how the cognitive system arbitrates between automatic and controlled pitch codes as contextual demands increase. Demonstrating graded, individual-specific strategy switching would extend conflict-monitoring and resource-rational frameworks of cognitive control theories to a rich, ecologically valid domain. It would also inform pedagogical approaches to RP training by acknowledging heterogeneity in musicians’ control dynamics.

## Materials and methods

### Participants

Fifty university students (25 male, 25 female; age range: 18–34 years, *M* = 20.5, SD = 3.25) participated after providing written informed consent. Participants varied widely in their musical backgrounds, with years of private musical instruction ranging from 0 to 20 years (*M* = 10.6, SD = 5.58). Here, musical experience was defined as the number of years receiving private lessons in instrumental (piano, *N* = 37; guitar, *N* = 4; other monophonic instruments, *N* = 21) or vocal performance (*N* = 1), with some participants having overlaps, while general school-based music education was excluded. Participants were labeled S1 through S50 in ascending order of their absolute pitch (AP) test scores.

**Figure 1:**
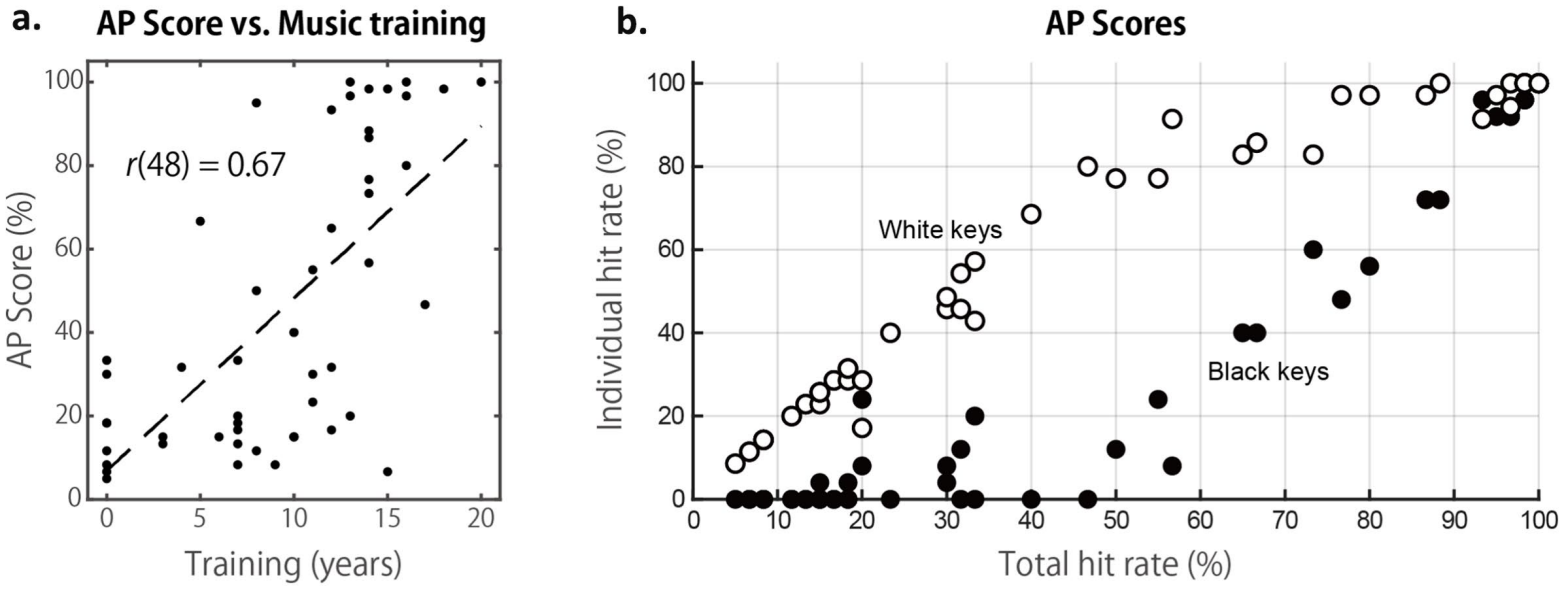
Summary of AP test results. **(a)** Scatter plot of AP scores plotted against years of musical training. **(b)** Accuracy for white-key (C, D, E, F, G, A, B) and black-key (C♯/D♭, D♯/E♭, F♯/G♭, G♯/A♭, A♯/B♭) notes, expressed as hit rates computed separately for each category, plotted against the overall hit rate (white-key plus black-key notes) in the AP test.

To minimize sampling bias, we randomly selected participants from a larger volunteer registry to approximate a broad, uniform distribution of musical experience and AP ability (Figure 1). Consequently, the final sample size (*N* = 50) was constrained by availability. Because our primary goal was to characterize error-pattern structure via cluster analyses—procedures not well served by standard *a priori* power formulas—this sample provided adequate precision to capture dynamics in cognitive strategies while remaining practical. All participants received monetary compensation. The protocol was approved by the Ethics Committee of Niigata University.

### Absolute pitch test

Participants completed an absolute-pitch (AP) identification task using 60 isolated piano tones (C2–B6) presented in random order, with the constraint that no pitch class was repeated on consecutive trials. Tones were delivered through Yamaha MSP7 Studio loudspeakers at a constant level, with a 5 s inter-stimulus interval. Using the fixed-Do solmization system, participants named the pitch class (e.g., Do, Re♯), ignoring octave. Responses were given orally and recorded by the experimenter. AP score was the percentage of correctly identified pitch classes.

### Relative pitch task (main task)

**Figure 2:**
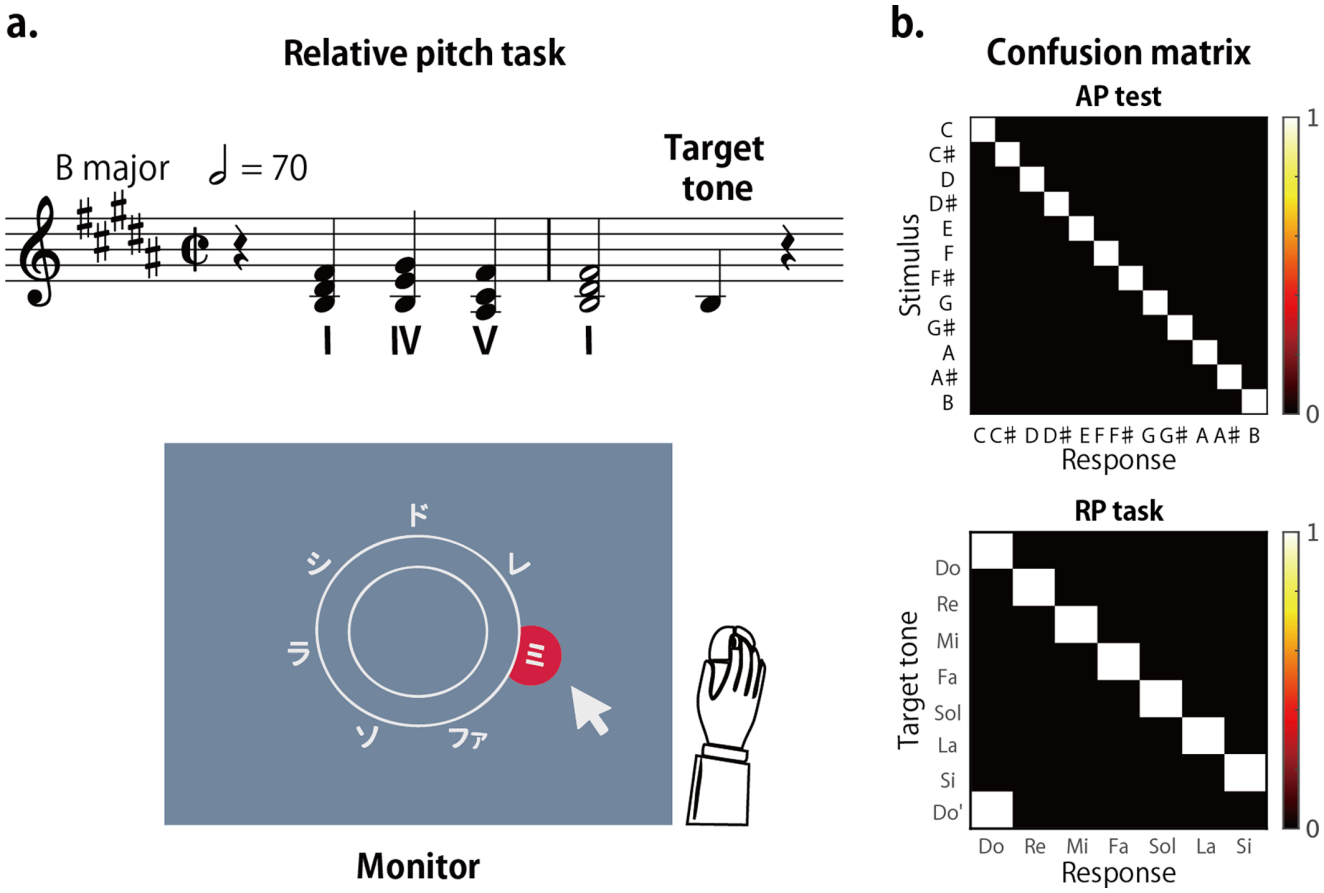
Experimental settings. **(a)** Each trial began with a four-chord progression (I–IV–V–I) establishing a major key, followed by a target tone. Participants identified the scale degree of the target tone using movable-Do solmization (Do, Re, Mi, Fa, Sol, La, Si). The key of the chord progression varied by condition: CM (C-major fixed), BM (B-major fixed), and RM (random major key). The musical notation in **(a)** shows an example from the BM condition. Response options were displayed in a circular layout on a computer screen, and participants selected their answer by clicking with the mouse within a 2.5 s response window after the target tone onset. **(b)** Confusion matrices for a perfect observer. The ordinate represents the presented stimuli, and the abscissa represents the participant’s responses.

Participants performed a relative pitch task in which they heard a four-chord progression (I–IV–V–I) establishing a major key, followed by a single target tone (Figure 2a). Although pitch identification of musical segments involves diverse mental, and sometimes visual, strategies that do not neatly fall into AP or RP categories (Letailleur et al., 2020), the authentic-cadence chord progression presented in this experiment encouraged participants to focus on the pitch relationships and rely on RP. This is because this particular chord progression provides one of the strongest cues for determining the tonic and the underlying musical scale from which the chords are built (Krumhansl, 1990; Piston and DeVoto, 1987). The tempo was set to 70 beats per minute, and the four-chord progression lasted approximately 2,200 ms, with the first three chords as quarter notes and the final chord as a half note. The target tone was a quarter note (~250 ms duration). The target tone was one of Do, Re, Mi, Fa, Sol, La, Si, or Do’, with Do’ being an octave-higher Do.

The participants were asked to identify the scale degree of the target tone using movable-Do solfège syllables (Do, Re, Mi, Fa, Sol, La, Si). For both Do and Do’, the correct solfège response was Do, although the stimulus pitch differed by one octave. Correct identification of Do’ as Do indicated the participant recognized octave equivalence. The participants were given 2.5 s to respond by clicking on a circular array of solfa syllables displayed on a computer screen in Japanese (Figure 2a). The time limit was determined in preliminary experiments to optimize the balance between participants’ performance and the overall experiment duration. The response options were arranged in a circular pattern to ensure equal distance to all choices and eliminate position-based reaction time biases. After each trial, participants manually returned the cursor to the center of the double circle so that every new trial started from the same central position, ensuring equal cursor-movement distance across all solfège options.

This task was administered under four conditions. In the C-major fixed (CM) condition, all trials were presented in the key of C major; in the B-major fixed (BM) condition, all trials were in B major. The Random major (RM) condition featured trials in randomly selected major keys from among all twelve, including C-major and B-major. In the Control condition, a spoken solfège syllable (e.g., “Mi”) was presented instead of the target tone, and participants identified the syllable they heard. All other aspects of the stimuli and task were the same as the other conditions. The voice recordings were from a native Japanese female speaker. Each condition comprised two blocks of 96 trials (192 trials per condition), with condition order randomized across participants. One condition lasted approximately 6.5 min, with short breaks between blocks and a longer break after completing the first four conditions. The entire experiment lasted about one hour.

Accuracy and reaction times (RTs) were recorded. Raw RTs were defined as the interval from target-tone onset to mouse click. Adjusted RTs for the CM, BM, and RM conditions were computed by subtracting each participant’s mean RT in the Control condition from their raw RTs in the CM, BM, and RM conditions. This adjustment isolated processing time specific to relative-pitch judgment by minimizing general perceptual and motor delays. The Control condition provided a stable baseline due to its near-perfect accuracy. Additionally, enforcing a common cursor-start position further reduced motor-related variability. Unless otherwise noted, all reported RTs refer to the adjusted RTs. Only correct trials were included when calculating the adjusted RTs to evaluate cognitive strategies underlying accurate responses.

The stimulus presentations and response recordings were controlled by a custom-made script running on PyscoPy version 2022.2.5 (Peirce et al., 2019). All stimuli were pre-recorded in WAV format using Cakewalk (version 2022.1) with high-quality sampled piano tones. Stimuli were presented through a Yamaha MSP7 Studio loudspeaker at a comfortable listening level.

### Error pattern analysis

To analyze error patterns, confusion matrices were computed for each participant and task, with the ordinate representing presented stimuli and the abscissa representing responses (Figure 2b). For the AP test, the matrix was 12 × 12, corresponding to the 12 pitch classes. For the RP task, the matrix contained 8 rows (Do, Re, Mi, Fa, Sol, La, Si, Do′) and 7 columns (Do–Si). Under perfect performance, both matrices would display a diagonal pattern; in the RP matrix, the diagonal would wrap around to reflect octave equivalence (Figure 2b). Trials without responses were excluded to focus on interpretable strategies. The overall proportion of excluded trials was 8.5%, leaving an average of 176 trials per condition for analysis.

We next grouped participants according to similarities in their error patterns using hierarchical clustering, a method that builds a tree showing which patterns are most alike. Before clustering, each confusion matrix was row-wise normalized, meaning we converted each row to proportions. We then converted each normalized matrix into a single list of numbers and compared these lists to determine how similar participants were to one another. Clustering was performed separately for each AP group (Low, Mid, High) and for each task condition (CM, BM, RM), using Euclidean distance as the metric. We first checked whether there was enough variation within each set by calculating the mean pairwise distance (MPD) between matrices; if MPD was below 0.1, the matrices were too similar for meaningful clustering. This occurred only in the High-AP group under the CM condition, where participants’ error patterns were nearly identical, so we treated that set as one uniformly similar cluster. For all other sets, we used the hierarchical clustering tree (the “dendrogram”) to decide how many clusters best captured the underlying structure.

### Data availability

All primary data, study materials (audio files and PsycoPy programs), and analysis scripts are publicly available.^1^

## Results

### Absolute pitch test

We first examined overall accuracy in the AP test, which ranged from 5%, which was close to the chance level performance of 1/12 = 8.3, to 100%. AP scores were positively correlated with years of musical training (Figure 1a), *r*(48) = 0.667, *p* < 0.001. At first glance, this may seem counterintuitive, as extended musical training alone does not guarantee AP acquisition, which is known to depend on musical training during a critical period ending around age 7 or 8 (Miyazaki and Ogawa, 2006). A likely explanation for this correlation is that our participants were mostly in their early twenties; thus, those with longer training histories (e.g., >15 years) likely began music training in early childhood, within the critical period for AP development, whereas those with shorter training histories did not.

Next, we calculated accuracy separately for white-key (C, D, E, F, G, A, and B) and black-key (C♯/D♭, D♯/E♭, F♯/G♭, G♯/A♭, A♯/B♭) notes (Figure 1b). This distinction was critical for the present study, as different major scales contain varying proportions of white- and black-key notes. For instance, the C-major scale comprises only white-key notes, whereas the B-major scale includes five black-key notes and only two white-key notes, which is the maximum ratio of the number of black-key to white-key notes in a key. Other non-C keys contain one to five black-key notes. Consequently, a listener’s AP accuracy for white- and black-key notes determines how effectively they can apply an AP strategy when naming pitches across different keys. For example, listeners whose AP is limited to white-key notes would rely on different strategies from those who can accurately identify both white- and black-key notes when processing B major or any other key that includes black-key notes.

As the total AP score increased from around the chance level to approximately 60%, the hit rate for white-key notes improved nearly linearly, while the accuracy of black-key notes remained low. Once the total score exceeded 60%, white-key accuracy reached a ceiling of over 80%, while black-key accuracy increased nearly linearly. This profile replicates the learning patterns reported in an earlier AP study (Miyazaki, 1988) and suggests a two-stage acquisition process, with white-key labels learned first to form the foundation for learning black-key labels. Thus, notes with accidentals (e.g., C♯ and D♯) and those without accidentals differ in how easily they can be identified in AP-based pitch perception. For convenience, we referred to these two categories as “white-key notes” and “black-key notes,” but this terminology is purely descriptive. We do not believe that the physical color of the piano keys plays any role in participants’ performance in the pitch naming tasks.

Based on the rationale that AP test performance for white-key and black-key notes should directly influence pitch-naming strategies in C-major and non-C tonal contexts, respectively, participants were divided into three groups according to their performance on these note types: Low AP, Mid AP, and High AP. The Low AP group (*N* = 12, S1–S11, S20) included those whose performance on white-key trials did not differ significantly from chance. With seven possible white-key notes (*p* = 1/7) and 35 white-key trials, the 5% chance-level criterion corresponded to a hit rate of 26%. The Mid AP group (*N* = 21, S12–S19, S21–S33) comprised participants who performed significantly above chance for white-key notes but below the criterion for black-key notes. With five possible black-key notes (*p* = 1/5) and 25 black-key trials, the 5% chance-level criterion was a hit rate of 36%. The High AP group (*N* = 17, S34–S50) included those who performed significantly above chance for both white-key and black-key notes.

**Figure 3:**
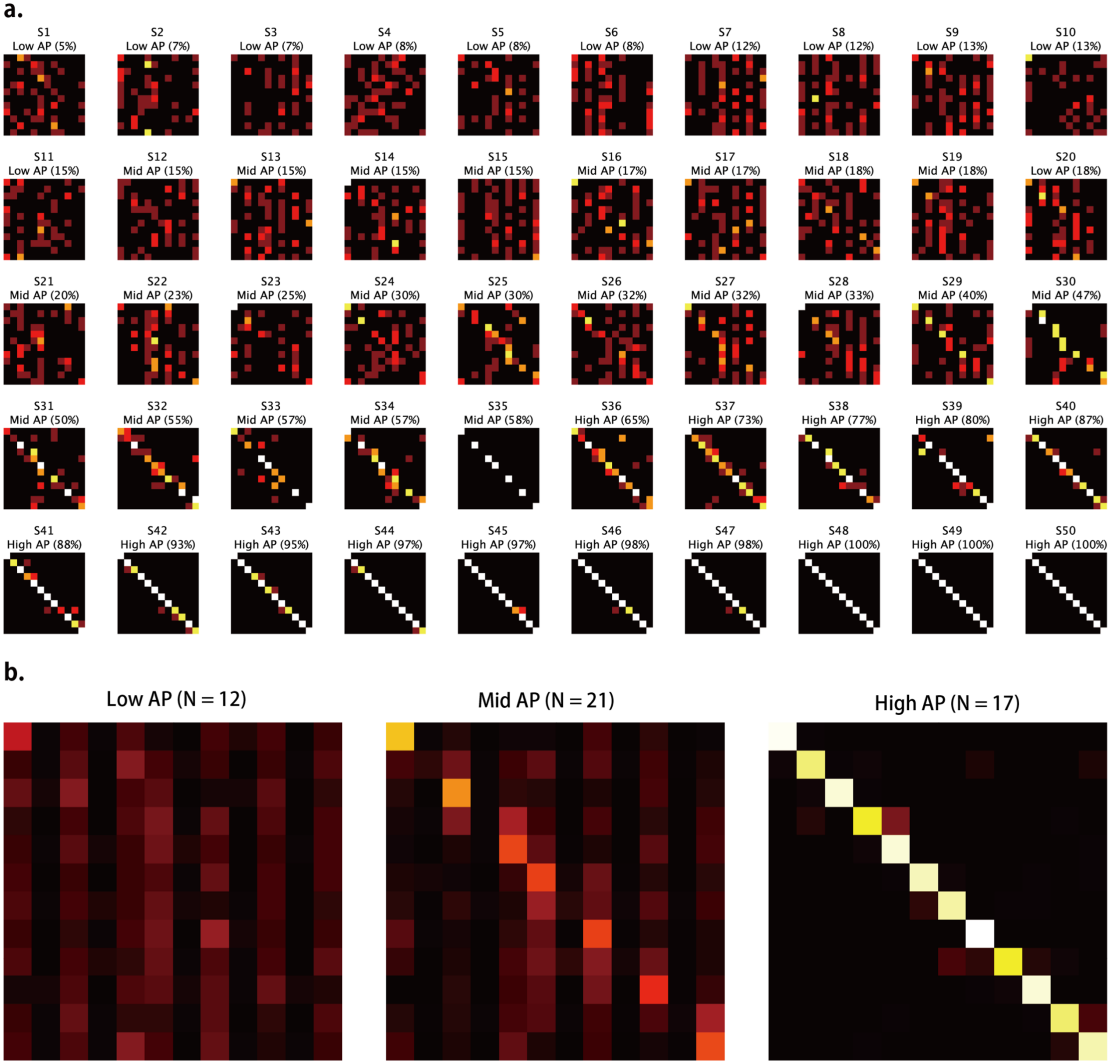
Confusion matrices for the AP test. **(a)** Individual confusion matrices (12 × 12) for each participant, in order of the AP test score. **(b)** Group-averaged confusion matrices for the three AP groups, classified based on their white- and black-key note performance.

Confusion matrices characterized the error structures of the three groups (Figure 3a). Participants with low AP produced matrices marked by dark vertical stripes in the black-key columns. This indicates that black-key labels were rarely chosen, regardless of the stimulus. Their remaining responses were widely dispersed across the matrix. At intermediate AP levels, errors clustered along the main diagonal. These listeners most often confused pitches separated by a few semitones, resulting in a fuzzy diagonal band. Participants with high AP levels generated a narrow, high-value diagonal, showing near-perfect identification of both white- and black-key tones. Group-averaged confusion matrices (Figure 3b) clearly depicted the contrasted error patterns of the three groups.

In summary, the findings indicate that AP ability varies along a continuum, with corresponding changes in the geometrical structure of error patterns reflecting pitch labeling accuracy for white-key and black-key notes. Three behaviorally distinguishable groups were identified based on the AP test: the Low AP group, who responded almost randomly and rarely selected black-key notes; the Mid AP group, who could reliably identify white-key notes but not black-key notes; and the High AP group, who could reliably identify both white-key and black-key notes. In the following sections, RP task data were analyzed separately for these AP groups, as appropriate.

### Relative pitch task (main task)

#### Hit rate and reaction time

We suppose that the distinct error patterns associated with different levels of AP are likely to influence cognitive strategies in RP tasks under varying key conditions, for the following reasons. In C major, where all scale tones are white-key notes, both High-AP and Mid-AP listeners can effectively use an AP-based fixed-Do solmization strategy. In contrast, in B major, where more than half of the scale tones are black-key notes, Mid-AP listeners would struggle with this strategy, as they cannot reliably identify black-key notes using AP. High-AP listeners, however, could still benefit from the fixed-Do approach, as they are able to identify the absolute pitch of black-key notes and convert these to moveable-Do labels by recognizing B as the tonic. This involves mentally shifting the fixed pitch label by one semitone to arrive at the correct response, albeit with increased cognitive load and reaction time. In the random-major condition, even High-AP listeners would find this strategy more challenging, as the tonic changes unpredictably and the on-the-fly transposition becomes more difficult.

**Figure 4:**
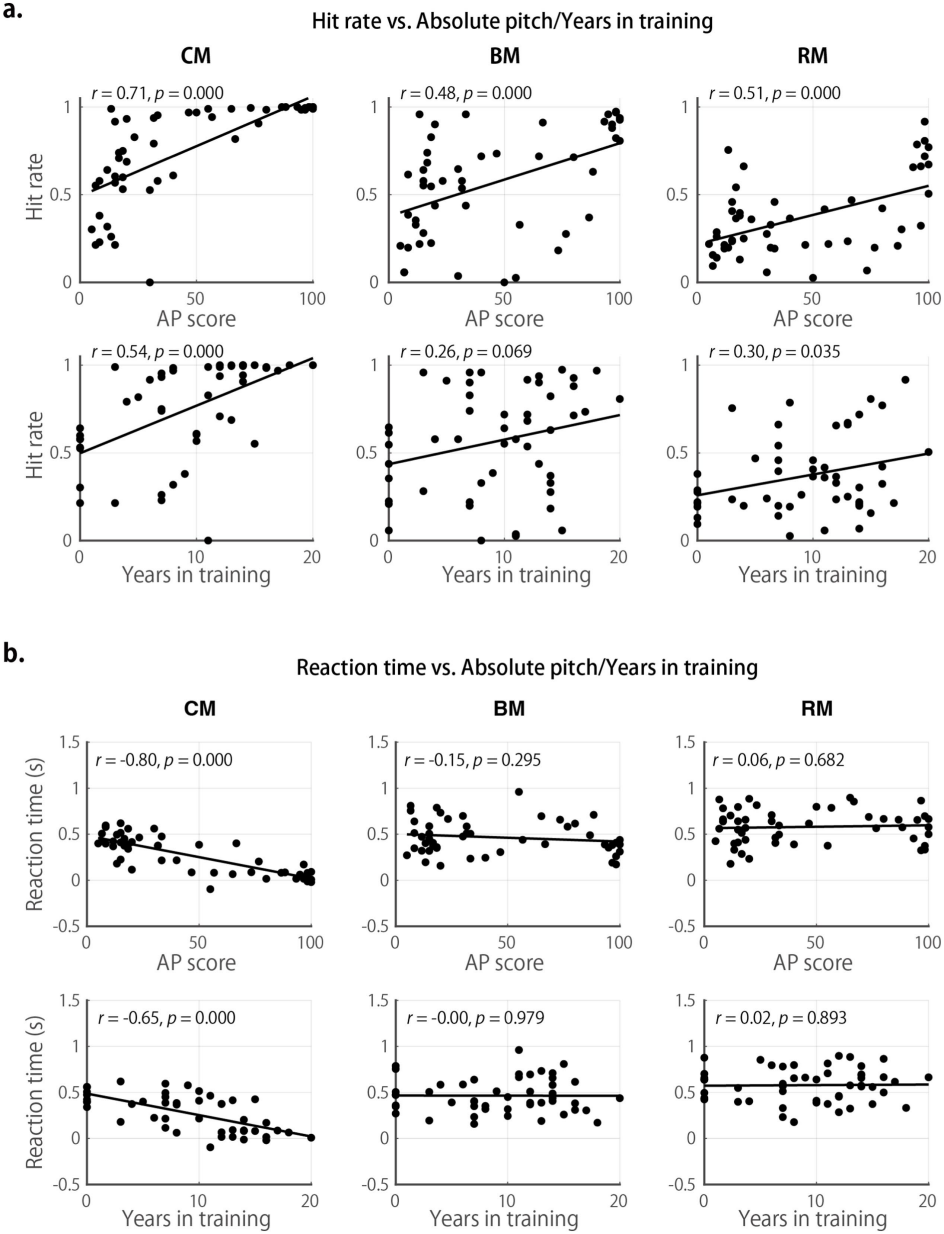
Effects of AP and years of musical training on the RP task performance. (**a**, upper row) Scatter plots showing the relationship between AP scores and accuracy (% correct) in the three RP task conditions: CM, BM, and RM. (**a**, lower row) Scatter plots showing the relationship between years of musical training and accuracy in the three conditions. (**b**, upper row) Scatter plots showing the relationship between AP scores and adjusted RTs (see Methods). (**b**, lower row) Scatter plots showing the relationship between years of musical training and adjusted RTs.

To test these predictions, we examined how AP scores influenced performance across the three RP tasks (Figure 4a). Scatter plot inspection suggested that overall accuracy was highest in the C-major fixed condition, followed by the B-major fixed condition, and lowest in the random-key condition. In all key conditions, RP scores were significantly positively correlated with AP scores, *r*(48)s > 0.477, *p*s < 0.001, indicating moderate trends (Evans, 1996). RP scores in all task conditions also correlated with years of musical training, which is expected given that RP scores were correlated with AP scores, and AP scores themselves were correlated with training duration (Figure 1). To disentangle these effects, we conducted a repeated-measures ANOVA with standardized AP scores and years of training as between-subject factors, and Task (CM, BM, RM) as a within-subject factor. This analysis revealed significant main effects of Task, *F*(2, 92) = 47.6, *p* (HF-corrected) < 0.001, *η*_p_^2^ = 0.509, and AP score, *F*(1, 46) = 15.5, *p* (HF) < 0.001, *η*_p_^2^ = 0.399, but no significant effect of years of training, *F*(1, 46) = 0.0, *p* (HF) = 0.830, *η*_p_^2^ = 0.002. None of the interactions reached significance (all *F*s < 1.3, *p*s (HF) > 0.264, *η*_p_^2^s < 0.029).

We next examined the RTs, computed as raw RTs in each task minus each participant’s mean RT in the Control condition. This baseline correction isolates the processing time specifically associated with pitch name identification. Notably, negative or near-zero RTs indicate that participants responded as quickly as, or even faster than, in the simple Control condition, suggesting highly automatic pitch labeling typical of AP. As shown in Figure 4b, in the C-major fixed condition, the RTs showed a strong negative correlation with AP scores, *r*(48) = −0.80, *p* < 0.001. Several high-AP participants exhibited negative RTs, demonstrating that for these individuals, pitch identification was so fast and automatic that it required no additional processing time beyond a simple motor response, and in some cases, even less. Negative (adjusted) RTs were possible because pitch perception can occur for sounds as brief as a few tens of milliseconds (Robinson and Patterson, 1995), whereas solfege syllable perception in the control task was constrained by the consonant–vowel structure, which extends over 100 ms and imposes a lower bound on processing time. This finding provides empirical support for the automaticity of absolute pitch processing in familiar tonal contexts.

Despite the advantage of AP in the C-major fixed condition, this merit of AP diminished in the B-major fixed condition and was absent in the random-key condition, with correlations of *r*(48) = −0.15, *p* = 0.295 and *r*(48) = −0.06, *p* = 0.682, respectively. A repeated-measures ANOVA revealed a significant interaction between AP score and Task, *F*(2, 88) = 6.3, *p* (HF) = 0.003, *η*_p_^2^ < 0.126, with no other significant main effects or interactions (all *F*s< 2.6, *p*s > 0.114, *η*_p_^2^s < 0.067). These findings suggest that in unfamiliar or unstable key contexts, AP listeners may rely on alternative strategies, such as pitch-name shifting or interval-based relative pitch, resulting in slower response times, a hypothesis explored further in the next sections.

**Figure 5:**
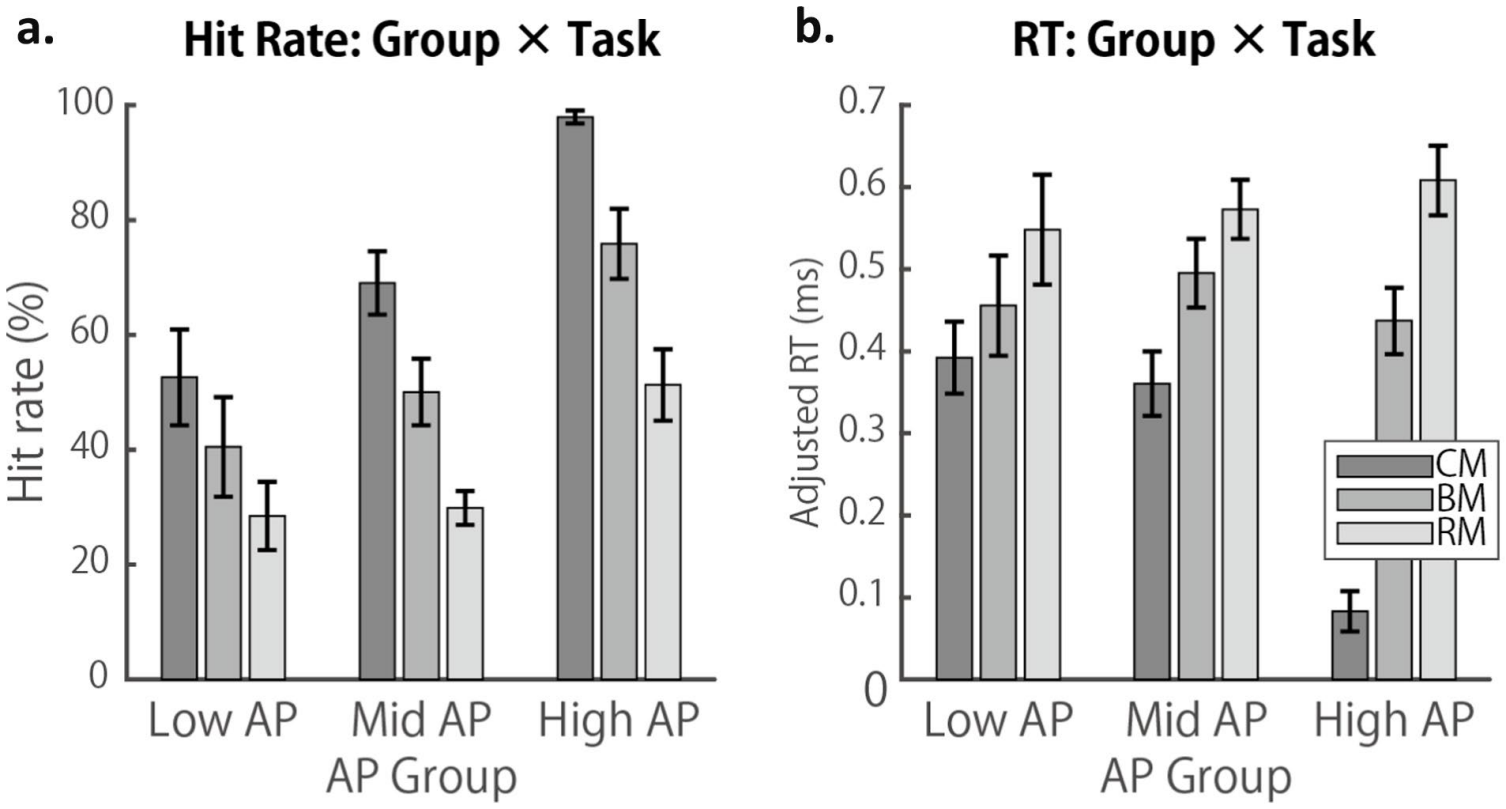
Group-based comparisons of RP task performance across AP levels. **(a)** Mean hit rate (% correct) in the three RP task conditions (CM, BM, and RM) plotted separately for low, mid, and high AP groups. **(b)** RTs averaged across correct trials in each RP task condition. Error bars represent SEM.

Group-based analyses were conducted to further examine the above findings, using the subject grouping derived from the AP test performance (Figure 5). ANOVA on hit rate data revealed significant main effects of Task, *F*(2, 94) = 60.4, *p* (HF) < 0.001, *η*_p_^2^ = 0.562, and Group, *F*(2, 47) = 13.7, *p* (HF) < 0.001, *η*_p_^2^ < 0.544, whereas the Group × Task interaction was not significant, *F*(4, 949) = 1.7, *p* (HF) = 0.164, *η*_p_^2^ < 0.068. *Post hoc* comparisons with Scheffé’s correction showed all pairwise task comparisons within each group were significant (*p*s < 0.01), except between CM and BM (*p* = 0.302) and BM and RM (*p* = 0.06) in the Low AP group. Although the nearly 50% hit rate of Low-AP participants in the C-major condition might appear high, this likely reflects the task design, in which the same key of C was repeated for the entire 6.5 min block, allowing participants to rely on RP once the tonal center became clear.

ANOVA on RT data yielded a significant main effect of Task, *F*(2, 90) = 49.0, *p* (HF) < 0.001, *η*_p_^2^ < 0.521, and Group × Task interaction, *F*(2, 94) = 8.7, *p* (HF) < 0.001, *η*_p_^2^ < 0.280. Post hoc comparisons with Scheffé’s correction indicated that all pairwise task comparisons within each group were significant (*p*s < 0.05), except for CM vs. BM (*p* = 0.610) and BM vs. RM (*p* = 0.236) in the Low AP group, and BM vs. RM in the Mid AP group (*p* = 0.444).

In summary, AP possessors benefited from their fixed pitch-labeling ability across all key conditions in terms of hit rate. However, both their hit rate declined and their reaction time increased from the C-major fixed condition to the B-major fixed and random-key conditions, indicating an increased cognitive load. Listeners with low AP proficiency also showed similar, though less pronounced, declines. The next section examines error patterns to investigate how participants with varying levels of AP adjusted their cognitive strategies in an effort to optimize performance.

#### Error pattern analysis

The analyses of hit rate and RT indicated that the RP task became increasingly difficult as the key condition changed from CM to BM and to RM, which was expected. We next analyzed the error patterns to reveal how the increase in cognitive load affected the cognitive strategy for solfa identification in listeners with various levels of AP.

**Figure 6:**
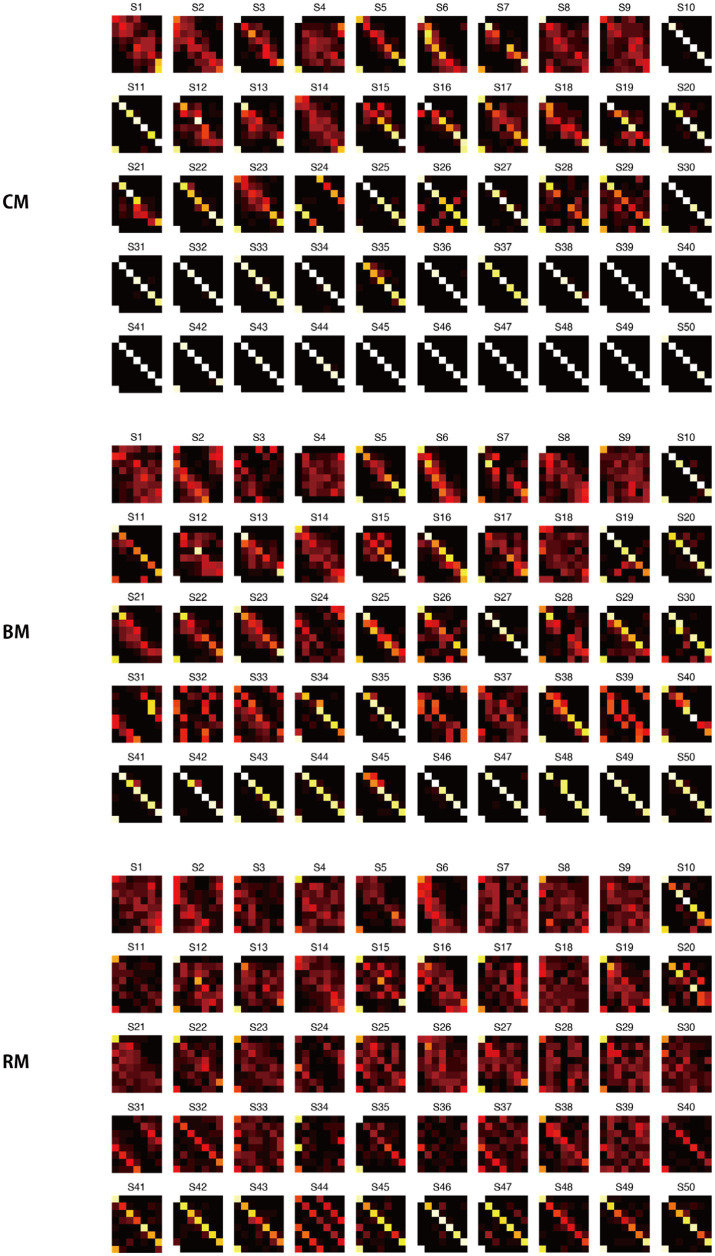
Individual confusion matrices for the RP task. Confusion matrices are shown for all participants under three task conditions: CM, BM, and RM. Stimuli (Do, Re, Mi, Fa, Sol, La, Si, Do′) are shown on the vertical axis, and participant responses (Do–Si) on the horizontal axis. Participants (S1–S50) are arranged in ascending order of AP scores.

Figure 6 presents the confusion matrices for all participants in the CM, BM, and RM conditions, with stimuli (Do, Re, Mi, Fa, Sol, La, Si, and Do′) shown on the ordinate and participant responses (Do, Re, Mi, Fa, Sol, La, and Si) on the abscissa. Several notable error patterns are evident:

- Nearly all notes were identified correctly (e.g., Participant S50: CM, BM, RM).
- Notes other than Do and Do′ were frequently confused with one another (e.g., Participant S4: CM, BM, RM).
- Notes other than the lower keynote (Do) were poorly identified (e.g., Participant S8: CM, BM).
- Notes other than Do, Fa, and Do′ were often confused with one another (e.g., Participant S12: CM, BM, RM).
- The interval from the lower keynote (Do) tended to be underestimated (e.g., Participant S6: CM, BM, RM).
- No scale tones were identified as Fa (e.g., Participant S7: CM, BM, RM).
- Notes were sometimes identified as being a fourth or fifth away from the stimulus (e.g., Participant S24: CM; S31: BM, RM).

Although some error patterns likely reflected idiosyncratic strategies unique to individual participants, others appeared to be shared across multiple individuals. To identify such commonalities, hierarchical cluster analysis was applied to the confusion matrices within each AP group (Low, Mid, and High), separately for the CM, BM, and RM conditions. The primary goal of this analysis was to examine how error patterns evolved as task difficulty increased from CM to BM and then to RM, thereby shedding light on changes in cognitive strategies across tasks in the different AP level groups.

**Figure 7:**
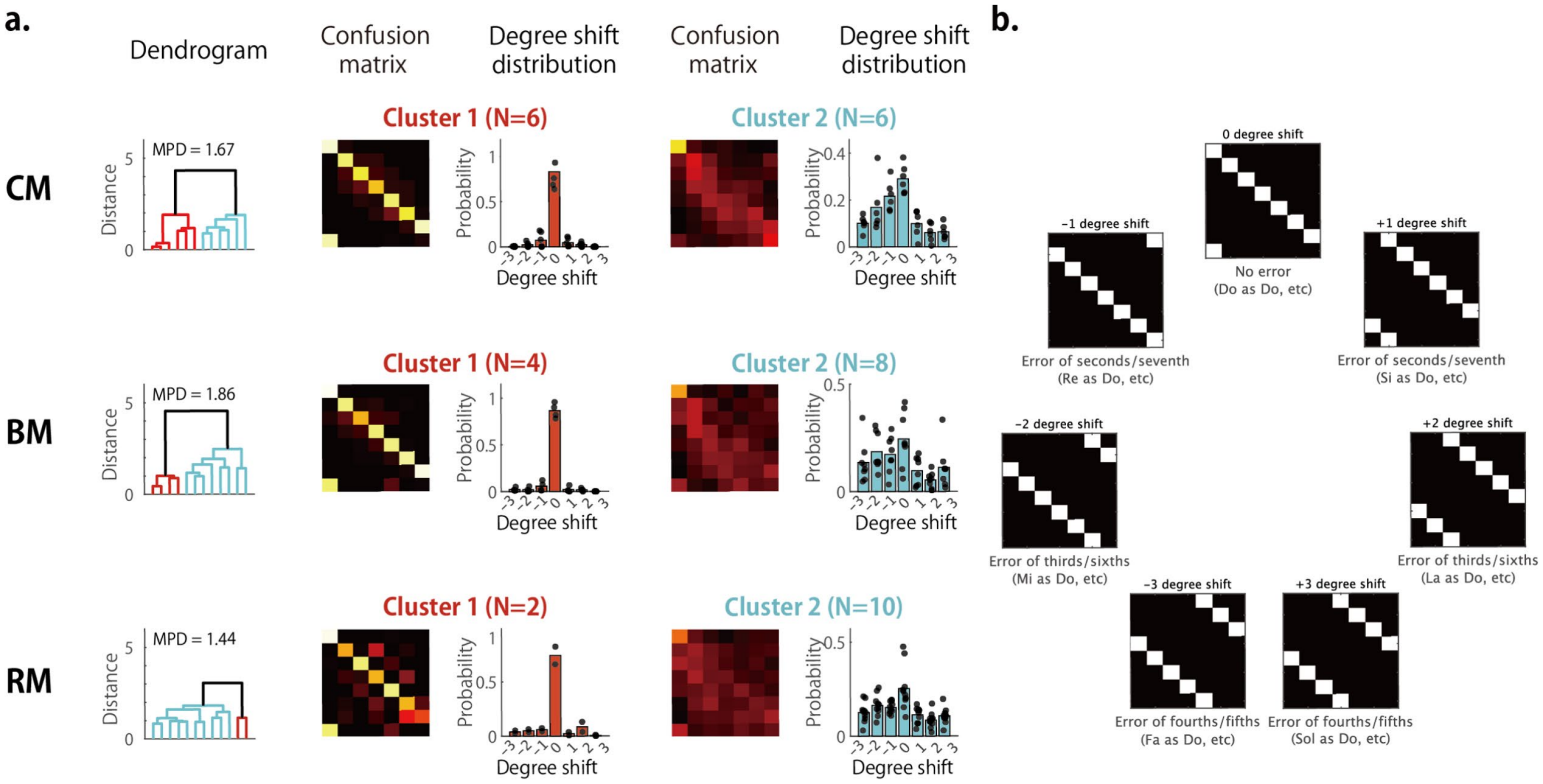
Cluster analysis of error patterns in the low-AP group. **(a)** Cluster-averaged confusion matrices in the low-AP group for the three RP task conditions: CM, BM, and RM. Participants were grouped into two clusters using hierarchical clustering. **(b)** Schematic examples of degree-shift distributions illustrating how systematic deviations appear in the analysis. These examples are not based on individual participant data but are intended to help interpret the shift distributions shown in **(a)**. MPD, mean pairwise distance.

In the Low-AP group, hierarchical clustering revealed two distinct response pattern profiles across all task conditions (Figure 7a). In the CM task, half of the participants (*N* = 6) produced a clear diagonal confusion matrix, indicating correct task execution (Cluster 1). The other half showed a characteristic error pattern (Cluster 2), in which the diagonal structure was unclearly articulated and responses exhibited systematic interval underestimation, with entries shifted toward the left of the diagonal. To quantify this interval deviations, cell counts were summed along the principal diagonal and successive sub-diagonals (Figure 7b), generating plots of degree-shift distributions displayed adjacent to each confusion matrix in Figure 7a. Cluster 1 displayed a pronounced peak at a zero-degree shift (correct responses), whereas Cluster 2 exhibited substantial occurrences of downward shifts of one degree or more, confirming a pervasive underestimation of intervals from Do. Another notable feature of Cluster 2 was that while the lower Do was identified accurately, the higher Do′ was often mislabeled as Si, signaling a breakdown of octave equivalence. Similar two clusters re-emerged in the BM and RM tasks. Across tasks, membership in Cluster 1 (accurate cluster) declined (CM > BM > RM), while membership in Cluster 2 (error-prone cluster) increased, reflecting increasing task difficulty.

**Figure 8:**
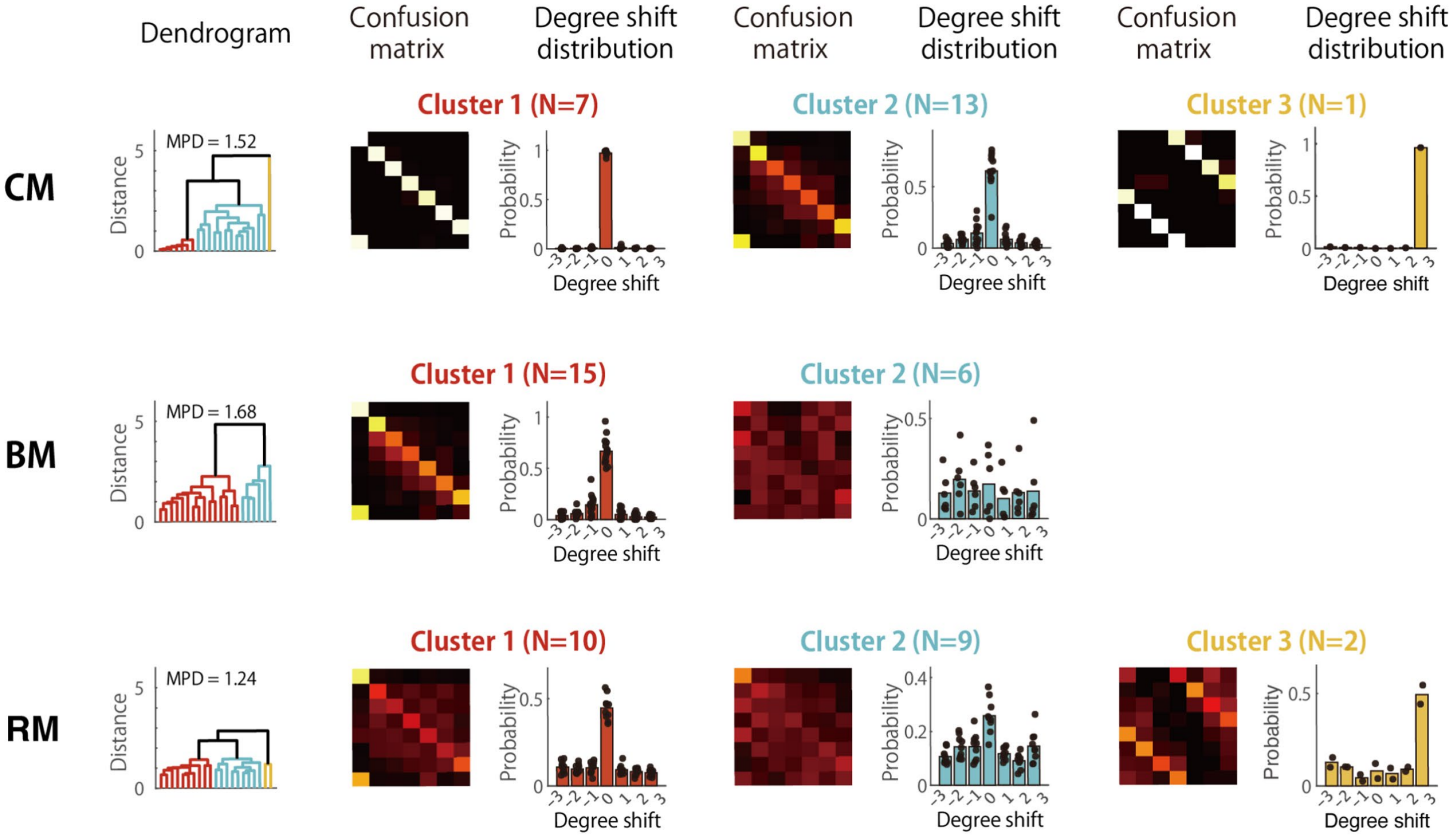
Cluster analysis of error patterns in the mid-AP group. Cluster-averaged confusion matrices in the mid-AP group for the three RP task conditions: CM, BM, and RM. MPD, mean pairwise distance.

Different error patterns emerged in the Mid AP group performing the same set of tasks (Figure 8). Clusters 1 and 2 in CM and Cluster 1 in BM and RM resembled Cluster 1 of the Low AP group, but a novel pattern appeared as Cluster 3 in CM and RM. This pattern was characterized by consistent errors of a fifth (or a fourth): Sol was misidentified as Do (a fifth below or a fourth above), Do and Do′ as Fa, and Re as La, etc. These systematic shifts displaced the diagonal of the confusion matrix rightward by three positions (or equivalently leftward by four), indicating a consistent intervallic error of fifths or fourths. Another distinctive pattern, seen in Cluster 2 under the BM condition, involved a leftward diagonal shift of two degrees. That is, Mi was misidentified as Do, Fa as Re, and so on, which are errors corresponding to thirds, and these errors produced the highest peak in the degree-shift distribution at −2 degrees, although there were individual differences. Notably, a similar −2 degree shift, though less pronounced, was also observed in the BM and RM conditions of the Low AP group and High AP group, where the second- or third-largest peak occurred at −2 degrees. Hence, errors of thirds were not uncommon. The occurrence of fifth- and third-interval errors suggests that participants relied on chord-component listening strategies, misidentifying the third (Mi) or fifth (Sol) of the final tonic chord as the keynote (Do) and then judging target tones according to their intervallic relation to that misidentified keynote.

**Figure 9:**
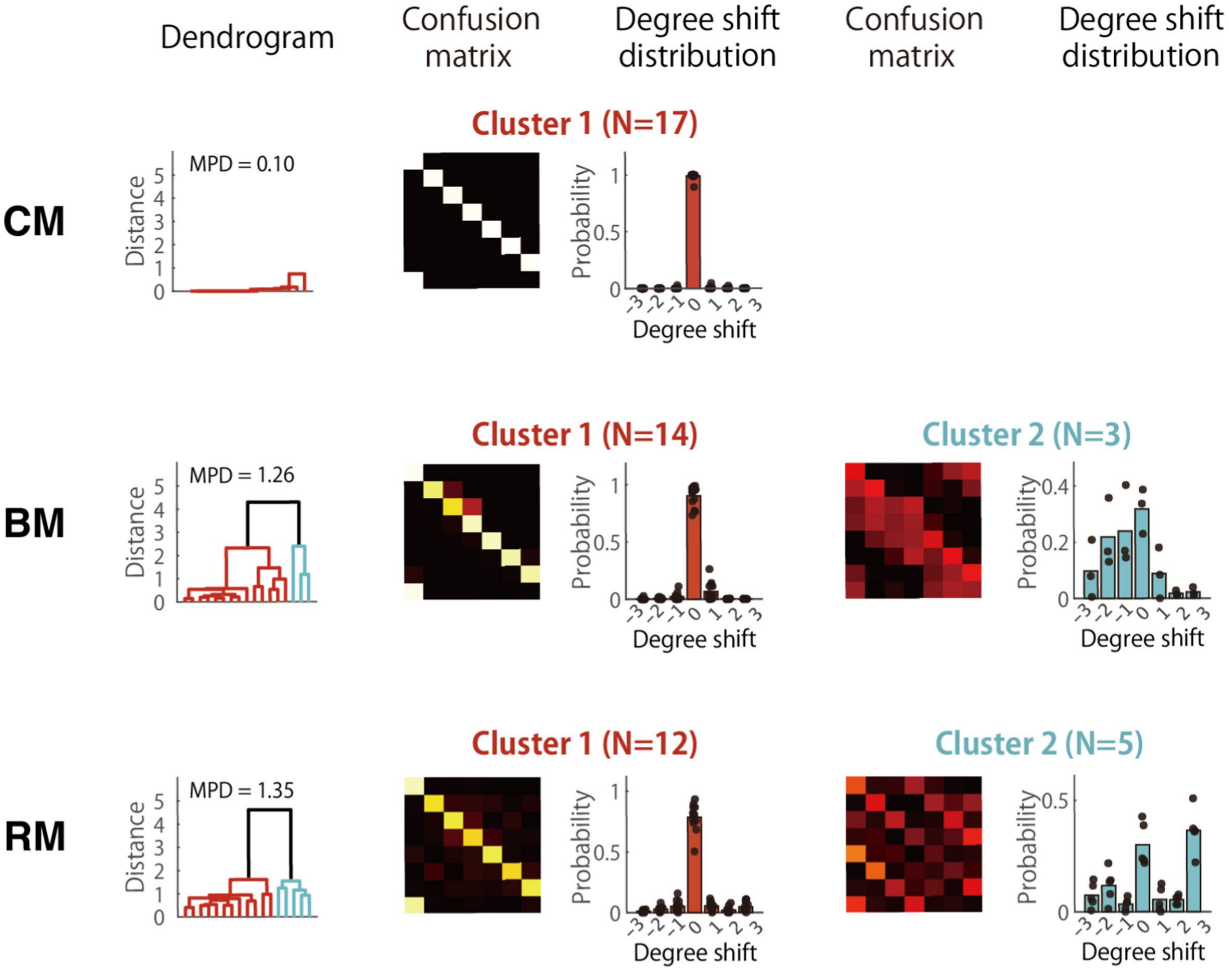
Cluster analysis of error patterns in the high-AP group. Cluster-averaged confusion matrices in the high-AP group for the three RP task conditions: CM, BM, and RM. Since the matrices were highly similar for all subjects in CM (MPD = 0.10), the result was interpreted as forming one coherent cluster. MPD, mean pairwise distance.

Distinct error patterns also emerged in the High AP group (Figure 9). In BM, the second cluster showed an asymmetric leftward shift of the diagonal by about one or two degrees: Do (i.e., having the absolute pitch of B) was misidentified as Si, Re (or C♯) as Do, Fa (or E) as Mi, and so on. This error pattern suggests failure to suppress the automated fixed labeling strategy. In the RM condition, however, this pitch-name shifting strategy in the High-AP group was apparently abandoned with the emergence of different error patterns, indicating errors of fifths or thirds (Cluster 2). This error pattern indicated that the participants misinterpreted any of the component tones of the tonic chord (i.e., Do, Mi, and Sol) as Do. The change in strategy from pitch-name shifting in BM to chord listening in RM was reasonable, as the random change of would make mental calculation of pitch shifting difficult and impractical.

To confirm this interpretation, we analyzed how the RTs of participants belonging to Cluster 1 in the High AP group changed with task. As a result, the mean RT was significantly prolonged from CM (83 ms) to BM (407 ms; *t*[29] = 6.7, *p* < 0.001, Cohen’s *d* = 2.51) and to RM (556 ms; [27] = 9.6, *p* < 0.001, *d* = 3.78), which suggested a strategic shift from direct and automatic fixed-Do labeling in CM to cognitively controlled use of AP for pitch-name shifting in BM and RM. Hence, the listeners with High AP in the RM condition either continued to use AP for pitch-name transpositions (Cluster 1) or changed their strategy to chord listening (Cluster 2). Since, under the RM condition, the mean AP score of subjects in Cluster 1 (*M* = 93%) was significantly higher than those in Cluster 2 (*M* = 80%), *t*[15] = 2.3, *p* = 0.035, *d* = 0.18, AP proficiency likely influenced their choice of strategy: Only listeners with the highest levels of AP persisted to exploit their AP in the most demanding task of RM.

## Discussion

Everyday cognition requires reconciling automatic, stimulus-bound codes with context-dependent goals, a hallmark challenge in cognitive control. Classic paradigms such as Stroop and flanker tasks index this reconciliation through color naming or spatial attention. Here we leveraged pitch perception as a real-life analogue to these paradigms: fixed-Do solmization based on AP provides a rapid, immutable label for a tone, whereas movable-Do solmization based on RP assigns pitch name labels based on the surrounding harmonic context. AP-based fixed-pitch labeling has been proposed to rely on specialized auditory cortical representations of pitch and pitch labels in the superior temporal gyrus (Itoh et al., 2005; Kim and Knösche, 2017), along with associative memory mechanisms in the left dorsolateral prefrontal cortex (Zatorre, 2003). In contrast, RP-based movable-Do labeling likely draws on AP-nonspecific pitch-processing and working-memory functions that are broadly shared across individuals (Itoh et al., 2005), although the precise neural mechanisms remain to be clarified. Transitioning between these qualitatively different modes of pitch naming therefore requires cognitive control to resolve conflict and reconfigure task strategy. Consequently, we treated AP strength as an individual-difference proxy for the baseline dominance of the automatic fixed-Do pitch labeling and asked how listeners along the AP proficiency continuum adapt their control strategies as conflict escalates in an RP task.

The fixed-Do and movable-Do pitch naming systems align perfectly in the key of C. However, these codes diverge when the tonal key shifts from C, creating a representational conflict akin to Stroop incongruence. Randomization of key further increase the cognitive load by making identification of the keynote (Do) difficult and by requiring trial-by-trial update of pitch name mapping from fixed-Do to movable-Do. Conflict-monitoring theories predict graded engagement of control: listeners with stronger automatic codes should incur larger interference costs and exhibit compensatory strategy shifts as conflict rises. Our findings overall confirmed this prediction, allowing music pitch perception to be successfully framed within the context of cognitive control theories. Furthermore, several novel insights were obtained regarding the interplay between automatic and controlled processes in pitch perception, as well as specific factors that trigger strategy switching.

In terms of hit rate and RT, the automatic pitch labeling in High-AP listeners was generally advantageous for pitch identification tasks, especially in low-conflict contexts such as the fixed C-major condition. However, this advantage diminished as tonal contexts deviated from C major. Both hit rate and RT data showed that the fixed-Do strategy became less efficient in the B-major condition and even more so under random key modulation, consistent with earlier findings on AP-related interference (Itoh et al., 2005; Miyazaki and Rakowski, 2002; Wilson et al., 2009). Under the cognitive demand incurred by the B-major condition, some high-AP participants showed error patterns suggestive of pitch-name transposition (e.g., systematic one-semitone shifts), a strategy that builds on their automatic fixed-Do labeling. In other words, they attempted to exploit their AP rather than abandoning it. Importantly, however, a completely different strategy of chord-component listening emerged in the random key condition (e.g., consistent errors of thirds or fifths), suggesting an abandonment of AP use. These errors are consistent with a chord-component listening strategy: the third and fifth were always present in the final chord of the sequence and thus provided strong cues for RP-based judgments. In other words, participants may have misidentified the tonic by attending to salient chord tones other than the tonic. Such confusions are plausible because, in a major key, the tonic, third, and fifth are perceptually more similar to one another than to other scale degrees (Krumhansl, 1990). Thus, as their default AP-based pitch-labeling system came into conflict with increasing task demands, AP musicians systematically recruited multiple alternative strategies.

Similar strategies for pitch labeling were observed in Mid-AP listeners but with an important difference: the chord-component listening strategy was seen in all task conditions, although it only appeared in the RM condition in the High AP group. This difference could be explained by the limited AP of Mid AP subjects, which enabled flexible strategy shift. Mid-AP listeners appear to occupy a cognitively unstable zone, where their partially reliable fixed-labeling leads to increased variability of alternative strategies of using it or abandoning it. In other words, a repertoire of representations are deployed flexibly in the absence of a single dominant code through metacognitive strategy selection.

Low-AP listeners mostly used RP-based strategies throughout the tasks. Individual differences in this group were not around the choice of strategy but concerned proficiency of RP ability. Those with limited RP in this group developed a characteristic error profile (e.g., failure of octave equivalence or interval underestimation), suggesting a stable, albeit suboptimal, internal framework for pitch name identification.

Overall, the differentiated results of the three AP groups inform theories of cognitive control by demonstrating flexible strategy arbitration in a naturalistic musical context. The results align with the conflict-monitoring model (Botvinick et al., 2001) and resource-rational models (Shenhav et al., 2013), which propose that individuals dynamically balance automaticity and control based on task demands. Here, AP listeners shifted from fast, automatic pitch naming to slower, effortful strategies (e.g., transposition or interval calculation) when tonal context rendered their default responses unreliable, but the choice of alternative strategy depended on was determined by an intricate balance between AP proficiency, musical key, and individual differences in their metacognitive choice of persisting with the AP strategy. In a broader theoretical context, AP can be viewed as a form of absolute sensory judgment, sharing characteristics, though not strict equivalence, with perceptual skills such as color naming, synesthesia, and eidetic imagery (Di Stefano and Spence, 2024). These parallels raise the possibility that similar forms of cognitive control may operate in other domains where rapid absolute judgments interfere with slower, context-sensitive forms of perceptual evaluation.

As concerns the neural mechanisms for cognitive control, the anterior cingulate cortex (ACC) monitors representational conflict and signals when current processing is suboptimal (Botvinick et al., 2001; Shenhav et al., 2013). In AP listeners, a key change away from C major is expected to induce conflict between fixed-Do labels retrieved automatically and the pitch relationships required for movable-Do judgments. This conflict would be detected by the ACC, which would then generate a control-demand signal. Midfrontal theta oscillations, thought to implement this control signal, would coordinate adjustments in lateral prefrontal cortex, enabling the inhibition of inappropriate fixed-Do labels and the recruitment of a slower, RP-based strategy.

Our findings have direct implications for music pedagogy, since the diversity of strategy profiles identified here suggests that a onesize-fits-all approach to RP training is suboptimal. Ear training programs might be enhanced by diagnostics that classify students by their error patterns, allowing for tailored interventions that build on individual strengths and mitigate weaknesses. For example, high-AP listeners may benefit from practicing in frequently and randomly changing keys to reduce reliance on fixed pitch–label associations. Students who struggle to identify the tonic should begin by reliably identifying Do and Do’, as these anchor tones provide the framework for recognizing other scale degrees. For learners with idiosyncratic error patterns (e.g., consistent confusion of particular scale degrees), training can target those specific notes directly. Such adaptive and individualized training approaches are expected to make RP learning more efficient and effective.

Several limitations warrant consideration. First, although the sample size was sufficient for clustering and regression analyses, larger samples are needed to examine finer-grained subgroups or potential developmental trajectories. Second, all participants were non–music-major university students in Japan, which limits the generalizability of the findings to professional musicians and to individuals of other age groups or cultural backgrounds. Third, the RP task used only major-key contexts and a single presentation modality (piano tones via speaker); future work should explore whether similar strategy patterns hold for minor keys, other timbres, or vocal production tasks. Future research should incorporate neuroimaging to elucidate the neural mechanisms that mediate shifts between AP- and RP-based strategies.

Another limitation concerns the measurement of AP, which may have been affected by listeners’ RP ability. In our study, we randomized the order of test tones, a simple and effective method for reducing RP-based strategies by minimizing the sense of tonality. However, a concern remains that even if a participant has only partial AP (e.g., accurate identification of A only), the subsequent tone could be judged relative to that known pitch, thereby inflating the AP score. This influence arises from short-term pitch memory and is difficult to avoid because AP assessment necessarily involves presenting multiple tones sequentially. While Wengenroth et al. (2014) used an ingenious method of presenting a gliding-pitch stimulus before each probe tone, this approach does not fully eliminate the problem because the final pitch of the glide may still function as a reference if the listener can identify it absolutely. Thus, our AP test scores, like those in other studies, may still have been partially affected by RP processes.

In conclusion, by combining accuracy, reaction time, and confusion-matrix analyses, we found that AP proficiency was associated with distinct patterns of cognitive control in real-life musical task in Western tonal music system. Rather than classifying AP as either helpful or harmful for RP tasks, as previous music cognition studies have hypothesized, we show that listeners flexibly switch among multiple pitch processing strategies, depending on their AP proficiency and cognitive task demands. By situating musical pitch processing within control theory, this work advances our understanding of how highly automated expertise representations interact with adaptive, goal-directed behavior.

## Data Availability

The datasets presented in this study can be found in online repositories. The names of the repository/repositories and accession number(s) can be found at: OSF, https://osf.io/8nrb7/.

## Footnotes

1
https://osf.io/8nrb7/

## References

[ref2] Botvinick M. M. Braver T. S. Barch D. M. Carter C. S. Cohen J. D. (2001). Conflict monitoring and cognitive control. Psychol. Rev. 108, 624–652. doi: 10.1037/0033-295X.108.3.624, 11488380

[ref3] Brattico E. Tervaniemi M. Näätänen R. Peretz I. (2006). Musical scale properties are automatically processed in the human auditory cortex. Brain Res. 1117, 162–174. doi: 10.1016/j.brainres.2006.08.023, 16963000

[ref4] Deutsch D. (2013). “Absolute pitch” in The psychology of music. ed. Deutsch D.. 3rd ed (San Diego, CA, USA: Academic Press), 141–182.

[ref5] Di Stefano N. Spence C. (2024). Should absolute pitch be considered as a unique kind of absolute sensory judgment in humans? A systematic and theoretical review of the literature. Cognition 249:105805. doi: 10.1016/j.cognition.2024.105805, 38761646

[ref6] Elmer S. Rogenmoser L. Kühnis J. Jäncke L. (2015). Bridging the gap between perceptual and cognitive perspectives on absolute pitch. J. Neurosci. 35, 366–371. doi: 10.1523/JNEUROSCI.3009-14.2015, 25568128 PMC6605251

[ref7] Eriksen B. A. Eriksen C. W. (1974). Effects of noise letters upon the identification of a target letter in a nonsearch task. Percept. Psychophys. 16, 143–149. doi: 10.3758/BF03203267

[ref8] Evans J. D. (1996). Straightforward statistics for the behavioral sciences. Pacific Grove, CA, USA: Brooks/Cole.

[ref9] Greber M. Jäncke L. (2020). Suppression of pitch labeling: no evidence for an impact of absolute pitch on behavioral and neurophysiological measures of cognitive inhibition. Front. Hum. Neurosci. 14:585505. doi: 10.3389/fnhum.2020.585505, 33281584 PMC7688746

[ref10] Itoh K. Suwazono S. Arao H. Miyazaki K. Nakada T. (2005). Electrophysiological correlates of absolute pitch and relative pitch. Cereb. Cortex 15, 760–769. doi: 10.1093/cercor/bhh177, 15371294

[ref11] Keenan J. P. Thangaraj V. Halpern A. R. Schlaug G. (2001). Absolute pitch and planum temporale. NeuroImage 14, 1402–1408. doi: 10.1006/nimg.2001.0925, 11707095

[ref12] Kim S. G. Knösche T. R. (2017). On the perceptual subprocess of absolute pitch. Front. Neurosci. 11:557. doi: 10.3389/fnins.2017.00557, 29085275 PMC5649255

[ref13] Krumhansl C. L. (1990). Cognitive foundations of musical pitch. Pacific. Oxford, UK: Oxford University Press.

[ref14] Letailleur A. Bisesi E. Legrain P. (2020). Strategies used by musicians to identify notes’ pitch: cognitive bricks and mental representations. Front. Psychol. 11:1480. doi: 10.3389/fpsyg.2020.01480, 32733333 PMC7358308

[ref15] Levitin D. J. Rogers S. E. (2005). Absolute pitch: perception, coding, and controversies. Trends Cogn. Sci. 9, 26–33. doi: 10.1016/j.tics.2004.11.007, 15639438

[ref16] Matsuda M. Igarashi H. Itoh K. (2019). Auditory T-complex reveals reduced neural activities in the right auditory cortex in musicians with absolute pitch. Front. Neurosci. 13:809. doi: 10.3389/fnins.2019.00809, 31447632 PMC6691098

[ref9001] Millera E. K. Cohen J. D. (2001). An integrative theory of prefrontal cortex function. Annual Review of Neuroscience, 24:167–202. doi: 10.1146/annurev.neuro.24.1.167, 11283309

[ref17] Mito H. (2003). Influence of absolute pitch on performance of sight-reading in transposed conditions. Jpn. J. Psychol. 74, 547–553.

[ref9002] Miyazaki K. (1995). Perception of relative pitch with different references: some absolute-pitch listeners can’t tell musical interval names. Perception & psychophysics, 57, 962–970. doi: 10.3758/bf03205455 8532499

[ref18] Miyazaki K. (1988). Musical pitch identification by absolute pitch possessors. Percept. Psychophys. 44, 501–512. doi: 10.3758/BF03207484, 3200669

[ref19] Miyazaki K. Ogawa Y. (2006). Learning absolute pitch by children: a cross-sectional study. Music. Percept. 24, 63–78. doi: 10.1525/mp.2006.24.1.63

[ref20] Miyazaki K. Rakowski A. (2002). Recognition of transposed melodies by absolute pitch possessors. Music. Percept. 20, 63–82. doi: 10.1525/mp.2002.20.1.63 12519030

[ref21] Oechslin M. S. Meyer M. Jäncke L. (2010). Absolute pitch--functional evidence of speech-relevant auditory acuity. Cereb. Cortex 20, 447–455. doi: 10.1093/cercor/bhp113, 19592570 PMC2803739

[ref22] Peirce J. W. Gray J. R. Simpson S. MacAskill M. R. Höchenberger R. Sogo H. . (2019). PsychoPy2: experiments in behavior made easy. Behav. Res. Methods 51, 195–203. doi: 10.3758/s13428-018-01193-y, 30734206 PMC6420413

[ref23] Piston W. DeVoto M. (1987). Harmony. 5th Edn. New York, NY, USA: W. W. Norton & Company.

[ref9005] Robinson K. Patterson R. D. (1995). The stimulus duration required to identify vowels, their octave, and their pitch chroma. Journal of the Acoustical Society of America, 98, 1858–1865. doi: 10.1121/1.414405, 22359341

[ref9003] Schlaug G. Jäncke L. Huang Y. Steinmetz H. (1995). In vivo evidence of structural brain asymmetry in musicians. Science, 267, 699–701. doi: 10.1126/science.7839149, 7839149

[ref24] Schulze K. Mueller K. Koelsch S. (2013). Auditory Stroop and absolute pitch: an fMRI study. Hum. Brain Mapp. 34, 1579–1590. doi: 10.1002/hbm.22010, 22359341 PMC6870281

[ref25] Sharma V. V. Thaut M. Russo F. A. Alain C. (2021). Neural dynamics of inhibitory control in musicians with absolute pitch. Cereb. Cortex Commun. 2:tgab043. doi: 10.1093/texcom/tgab043 34514414 PMC8423588

[ref26] Shenhav A. Botvinick M. M. Cohen J. D. (2013). The expected value of control: an integrative theory of anterior cingulate cortex function. Neuron 79, 217–240. doi: 10.1016/j.neuron.2013.07.007, 23889930 PMC3767969

[ref27] Stroop J. R. (1935). Studies of interference in serial verbal reactions. J. Exp. Psychol. 18, 643–662. doi: 10.1037/h0054651

[ref28] Takeuchi A. H. Hulse S. H. (1993). Absolute pitch. Psychol. Bull. 113, 345–361. doi: 10.1037/0033-2909.113.2.345, 8451339

[ref29] Temperley D. Marvin E. W. (2008). Pitch-class distribution and the identification of key. Music. Percept. 25, 193–212. doi: 10.1525/mp.2008.25.3.193

[ref30] Wengenroth M. Blatow M. Heinecke A. Reinhardt J. Stippich C. Hofmann E. . (2014). Increased volume and function of right auditory cortex as a marker for absolute pitch. Cereb. Cortex 24, 1127–1137. doi: 10.1093/cercor/bhs391, 23302811

[ref31] Wilson S. J. Lusher D. Wan C. Y. Dudgeon P. Reutens D. C. (2009). The neurocognitive components of pitch processing: insights from absolute pitch. Cereb. Cortex 19, 724–732. doi: 10.1093/cercor/bhn121, 18663250 PMC2638817

[ref32] Zatorre R. J. (2003). Absolute pitch: a model for understanding the influence of genes and development on neural and cognitive function. Nat. Neurosci. 6, 692–695. doi: 10.1038/nn1085, 12830161

[ref33] Zatorre R. J. Perry D. W. Beckett C. A. Westbury C. F. Evans A. C. (1998). Functional anatomy of musical processing in listeners with absolute pitch and relative pitch. Proc. Natl. Acad. Sci. USA 95, 3172–3177. doi: 10.1073/pnas.95.6.3172, 9501235 PMC19714

